# Risk Factors for Lung Cancer in the Province of Lecce: Results from the PROTOS Case–Control Study in Salento (Southern Italy)

**DOI:** 10.3390/ijerph19148775

**Published:** 2022-07-19

**Authors:** Fabrizio Minichilli, Francesca Gorini, Giovanni De Filippis, Elisa Bustaffa, Anna Maria Raho, Anna Melcarne, Fabrizio Quarta, Giuseppe Maggiore, Adele Idolo, Francesca Serio, Tiziana Grassi, Francesco Bagordo, Idelberto Francesco Castorini, Giovanni Imbriani, Fabrizio Bianchi, Prisco Piscitelli

**Affiliations:** 1 Institute of Clinical Physiology, National Research Council, Via Moruzzi 1, 56123 Pisa, Italy; francesca.gorini@ifc.cnr.it (F.G.); elisa.bustaffa@ifc.cnr.it (E.B.); fabriepi@ifc.cnr.it (F.B.); 2 Local Health Authority ASL Lecce, Via Miglietta 5, 73100 Lecce, Italy; giov.defilippis@gmail.com (G.D.F.); annamaria.raho@gmail.com (A.M.R.); melcarneanna@gmail.com (A.M.); uose@asl.lecce.it (F.Q.); giuseppe.maggiore@yahoo.it (G.M.); adeleidolo73@gmail.com (A.I.); francesco.castorini@gmail.com (I.F.C.); repol@ausl.le.it (P.P.); 3 Department of Biological and Environmental Sciences and Technologies, University of Salento, Via Monteroni 165, 73100 Lecce, Italy; francesca.serio@unisalento.it (F.S.); tiziana.grassi@unisalento.it (T.G.); francesco.bagordo@unisalento.it (F.B.); giovanni.imbriani@unisalento.it (G.I.)

**Keywords:** lung cancer, cigarette smoking, occupational exposure, environmental exposure, matched case–control study

## Abstract

In the province of Lecce (southern Italy), a higher incidence of lung cancer (LC) among men compared to regional and national data was reported. In a sub-area in the center of the province (cluster area), the incidence and mortality for LC was even higher. PROTOS is a case–control study aimed at investigating possible risk factors for LC in the province area. A total of 442 patients with LC and 1326 controls matched by sex and age living in the province of Lecce for at least 10 years were enrolled and georeferenced; they filled in a questionnaire with their personal information and exposures. For each risk factor, an Odds Ratio adjusted for all the other variables was calculated. The risk of LC increased with excessive use of alcohol in women, for those subjects with a family cancer history, for each increase in pack/year of cigarettes, for men more exposed considering the industrial district in the cluster area, and for those using pesticides in agriculture without wearing personal protective equipment. The higher incidence of adenocarcinoma in both sexes suggests that, in addition to cigarette smoking, concurrent exposures to other environmental, occupational, and life-style factors may play a role in increased cancer risk and should be more deeply explored.

## 1. Introduction

**Figure 1:**
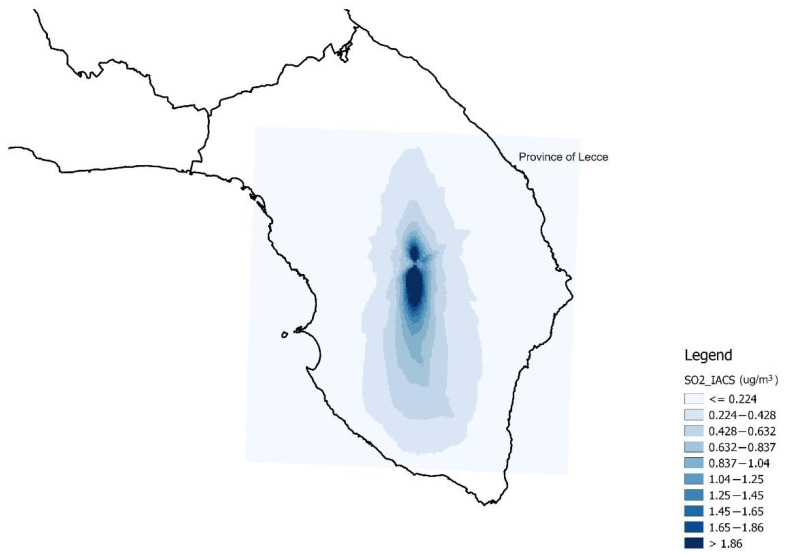
Spatial distribution of sulfur dioxide concentrations emitted by the plants included in Industrial Area of Central Salento (IACS) (SO_2__IACS) (data in µg/m^3^ from 1990).

Lung Cancer (LC) represents the second leading cause of cancer-related deaths at the global level, being responsible for approximately 2.2 million new cases per year (11.4% of all tumors) and nearly one death out of five cancer deaths [1]. According to international surveillance figures [1], LC among men is the most frequently diagnosed cancer (data from cancer registries of 36 countries) and the leading cause of cancer death (records from 93 countries). Up to 90% of global LC cases are attributed to the carcinogens related to active or passive tobacco smoking [2,3]. Over the few last decades, the epidemiology of LC has considerably changed in terms of incidence by sex, age group, and histological type [4]. Over the past 30 years, the incidence rate of adenocarcinoma has risen so rapidly that it has become more frequent than the squamous cell carcinoma, which was the most common histological type associated with cigarette smoking [5]. In many high-income countries, increasing incidence rates of LC have been observed in young women at rates higher than in men, a phenomenon partially explained by changes in smoking habits [6,7]. Although the majority of LC occurs in smokers, less than 20% of smokers develop LC, thus indicating a possible multi-factorial etiology or exposure to concurrent factors capable of substantially increasing the risk [8]. Indeed, a relevant proportion (10–20%) of LC cases, especially in women, is attributable to causes other than cigarette smoking [9].

In addition to smoking, environmental and occupational exposures to various types of hazardous substances, as well as genetic susceptibility, represent possible risk factors for LC in non-smokers [10]. The occurrence of LC in non-smokers presenting a family history of LC supports the hypothesis of an association between a hereditary component and the risk of lung carcinoma [10]. In fact, subjects with family history of cancer among first-degree relatives have a 50% higher risk of developing LC than those without familiarity, even after adjusting for smoking and other potential confounders [11]. Among environmental factors, radon—a radioactive gas originating from the decay of uranium and thorium in soil and rocks—has been classified as a human carcinogen by the International Agency for Research on Cancer (IARC), and it is considered the second-most important risk factor for LC after tobacco [3], although a very heterogeneous environment distribution must be considered. Outdoor air pollution is currently the main environmental hazard to human health, especially for the respiratory system [12]. Globally, air pollution is estimated to have caused 4.2 million deaths in 2016, including 29% attributable from LC [13], while 307,680 LC deaths in 2019 were attributed to ambient PM2.5 [14]. Nitrogen oxides (NOx), benzene, and particulate matter (PM10, PM2.5, PM1) are mainly attributable to vehicular traffic, whilst thermal power plants, home heating systems, and diesel-engines are the main sources of sulfur dioxide (SO_2_) [15,16].

LC has also been linked to different occupational exposures, with strong levels of evidence [17]. Occupational determinants known to increase the risk of pulmonary tumors include exposure to asbestos, ceramic, and construction industries; metal productions (particularly plants producing certain metals such as hexavalent chromium, nickel, cadmium, arsenic, and beryllium); shipbuilding; glass factories; coal mining; chimney sweeping; and painting [17,18]. The relationship between asbestos exposure and LC is clearly linear with no apparent threshold, and a current consensus view is that all types of asbestos fibers are carcinogenic. Furthermore, tobacco smoking synergistically interacts with asbestos, with a multiplicative pro-carcinogenic effect for the development of LC and mesothelioma [19].

Regarding individual lifestyles, it has been reported that alcohol, classified as a group I carcinogen [20], is associated with a 15% increased risk of LC in heavy drinkers compared to non-drinkers or occasional drinkers [21]. Additionally, alcohol consumption was associated with a modest increase in risk for lung carcinoma and adenocarcinoma in the highest category (≥7 drinks/day) of consumption [22]. Dietary factors may also influence the development and progression of LC [10]. In particular, the highest category of red meat consumption versus the lowest was positively related to an increased risk of LC by approximately 35% [23,24] due to high levels of saturated fat and the formation of carcinogenic heterocyclic amines as well as polycyclic aromatic hydrocarbons in meats cooked at elevated temperatures [23].

Since the 1970s, the province of Lecce (an area with 831,000 inhabitants located in the Salento peninsula, Apulia region, southern Italy) has shown higher incidences of and mortality rates for LC among men than those recorded at both the regional and national levels [25,26,27,28]. In the Lecce province, the absence of industrial districts comparable to those present in the neighbor areas of Brindisi and Taranto, as well as the proportion of smokers in line with that of the other provinces or even lower [25,29], is worth consideration.

The Italian National Institute of Health (ISS) has identified a “cluster area” with an excess of incidence of LC in men, involving 16 municipalities of the central area of Lecce province (355 observed LC cases vs. 285 expected) [30].

The case–control study PROTOS (Pulmonary cancer and Risk factors for Tumors, Observational Study) was set up in the framework of a specific Cancer Prevention Network in the province of Lecce (RePOL). The specific objective of this study was to investigate the associations between LC in the province of Lecce and all possible risk factors, with particular attention being paid to determinants related to occupational and environmental exposures.

## 2. Methods

PROTOS was designed as an observational study, with cases and controls matched by sex and age (five-year age groups) and enrolled via different channels (hospital/ambulatories or commissions for the granting of disability allowance). The ratio between cases and controls was set at 1 to 3 for both men and women. According to data from the Cancer Registry of the Local Health Authority (ASL Lecce), it was estimated that about 500 new cases per year was the incidence of LC in the province of Lecce. Therefore, we decided to perform up to 500 interviews by using a questionnaire aimed at obtaining information on risk factors for LC—sent to patients with LC diagnosed between 1 July 2015 and 31 December 2016, confirmed by histological diagnosis. The enrolled subjects were patients with LC and controls living in the province of Lecce for at least 10 years and were all asked to sign an informed consent form (including privacy) at the time of enrolment. 

The exclusion criteria for control subjects were established considering conditions potentially influenced by the same risk factors for LC: personal history of cancer at any site, asthma, emphysema, chronic obstructive pulmonary diseases, diabetes, thyroid or kidney diseases, intestinal malabsorption, myocardial infarction, heart failure, coronary artery diseases, stroke or cerebral-vascular accidents and other vascular conditions, and autoimmune diseases including psoriasis and multiple sclerosis. Patients also suffering from deafness, severe hearing loss or dementia, psychosis, or other neuro-degenerative diseases (supposed not to be able to cooperate during the interviews) were not admitted to the study as control subjects.

### 2.1. Recruitment

The interviews were carried out on a voluntary basis with patients with LC (cases) at the local Civil Invalid Commissions (CICs) for the granting of disability allowance and at hospital divisions of Radiotherapy, Thoracic Surgery and Oncology of Lecce (Fazzi Hospital), Casarano (Ferrari Hospital), Gallipoli (Sacred Heart Hospital), and Tricase (Cardinal Panico Foundation). A histological diagnosis confirming the malignancy of LC was obtained for each patient. The interviews of the control subjects were carried out on a voluntary basis involving patients who were visited at the same local CICs due to issues not related to neoplasms (usually people suffering from impaired vision or osteo-articular problems) or patients who were visited at the hospital divisions of Ophthalmology, Otolaryngologist, Orthopedics, and Plastic Surgery.

There are 26 CICs covering the entire province of Lecce with a considerable level of granularity (1 CIC per 40,000 inhabitants), thus excluding distortions due to distance (affecting the willingness to travel for control conditions compared to LC). Moreover, almost all people with various kinds of disabilities, deficits, or conditions/diseases apply for CIC visits to request financial support from the State (an allowance of EUR600–1200 per month); this “economic” incentive is a factor that pushes people to undergo medical examinations at the CICs. 

Cases were matched by sex and age ± 5 years with controls recruited from the same CIC (CIC recruitment channel) or hospital divisions/outpatient clinics (hospital/outpatient recruitment channel). The interviews were conducted (in about 15–20 min per patient) by properly trained staff belonging to the ASL Lecce, who also had the task of collecting informed consent and verifying the presence of exclusion criteria before starting the interview; this was conducted using a standardized check list.

### 2.2. Questionnaire

The PROTOS questionnaire was aimed at investigating individual risk factors for LC. The questionnaire was made up of 152 questions divided into 6 macro sections: (1) personal information; (2) behaviors and lifestyles; (3) personal and family clinical history; (4) residential history; (5) exposure to sources of air pollution; and (6) work and professional history. All filled questionnaires were first checked to exclude the incomplete and incorrect ones from the analyses, as well as to assess the presence of any positive response regarding any of the exclusion criteria.

### 2.3. Exposure Assessment

Table 1 shows the variables that were selected as proxy measures of both individual and environmental exposures potentially associated with LC. For some risk factors, the exposure assessment was more detailed as reported in the following paragraphs.

#### 2.3.1. Exposure to Tobacco Smoking

Smoking habit was defined as a categorical variable: non-smoker, former smoker, and current smoker CS. Exposure to tobacco smoke was also estimated through a continuous variable called Average Number of Packages Year of Cigarettes smoked (ANPYC) calculated as (average number of cigarette per day × years of active smoking)/20. Smoking habits and ANPYC variables were collinear with each other, and therefore we decided to use the variable ANPYC in the multiple regression model, as it was considered a more accurate proxy for smoking.

#### 2.3.2. Exposure Assessment to Industrial Plants and Occupational Factors

All cases and controls were georeferenced (the georeferenced residence was that of the period 1987–1997, i.e., the one compatible with the LC induction latency period) in order to perform specific analyses concerning the exposure to the two major emission sources of air pollutants, i.e., the Industrial Area of Brindisi (IAB), located on the northern border between the provinces of Lecce and Brindisi, including a chemical industry and a carbon-fired power plant (as the diffusion of its emissions also affect part of the study area in Lecce province) and the Industrial Area of Central Salento (IACS), located in the “cluster area”, with the presence of a big cement plant, a bitumen factory, and other small industrial chimneys with potential environmental impacts. Some of the industrial plants located in both these industrial areas were subject to specific Integrated Environmental Authorization (IEA) procedures from regional and local regulatory authorities, as well as periodic monitoring, with official controls performed by the Regional Environmental Protection Agency (ARPA Puglia). The dispersion models of the pollutants emitted by the two industrial areas were estimated using as a proxy of the models obtained by processing the data of the available IEAs (i.e., that of the chemical industry and fossil fuel-fired power plant located in Brindisi and that of the cement plant for the IACS). In particular, the dispersion models used SO_2_ and NO_x_, since they are considered by the scientific literature to be good proxies for the exposure to carbon-fired power plants [31,32,33] and to air pollution from industrial sources [34], including cement plants [35], respectively. The distributions of the average annual concentration of NO_x_ and SO_2_ on the ground related to the cement plant, estimated by the Institute of Atmospheric Sciences and Climate of the National Research Council of Lecce (CNR-ISAC) [36] through the RAMS-CALMET-CALPUFF system [37,38], were used as proxies for those of IACS. The emission scenario referred to the maximum emission limits established by Ministerial Decree no. 176 of 12/07/90 (emission data from 1990), and the reference meteorological data were those of 2005. The distributions of the average annual concentrations of NO_x_ and SO_2_ on the ground related to chemical industry and fossil fuel-fired power plants, estimated by ARPAP [39] using the SPRAY Lagrangian model [40,41], were used as proxies of those for IAB. This model simulated the deposition on the ground of pollutants considering the emission and meteorological data of 1993 and 2007, respectively.

**Figure 2:**
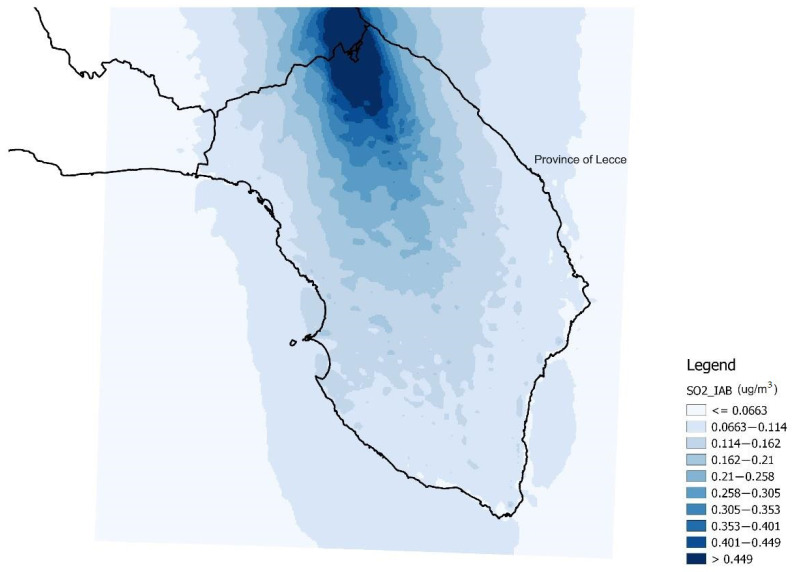
Spatial distribution of sulfur dioxide concentrations emitted by the plants included in the Industrial Area of Brindisi (IAB) (SO_2__IAB) (data in µg/m^3^ from 1993).

In order to have the same pollutants as proxies in the various analysis models, SO_2_ was used as a proxy of the IACS emissions (referring to 1990), which is strongly correlated with NO_x_ (ρ = 0.99, *p* < 0.001). Therefore, SO_2_ was used as proxy of atmospheric pollution produced by both IACS and IAB. Figure 1 shows the spatial distribution of SO_2_ emitted by IACS, and Figure 2 shows those emitted by IAB.

The individual exposure values to the atmospheric pollution produced by both IACS (expSO_2__IACS) and IAB (expSO_2__IAB) were defined by attributing to each individual the SO_2_ concentration estimated at the georeferenced residence. ExpSO_2__IAB shows a distribution with low variability and a maximum value <1 µg/m^3^ (Table 2).

The distribution of expSO_2__IACS has a higher average value than that of expSO_2__IAB, with greater variability and a maximum value of 5.4 µg/m^3^ (Table 2). The two distributions expSO_2__IACS and expSO_2__IAB are significantly correlated (ρ = −0.1465, *p* < 0.001) but not collinear each other (mean VIF = 1.06).

**Figure 3:**
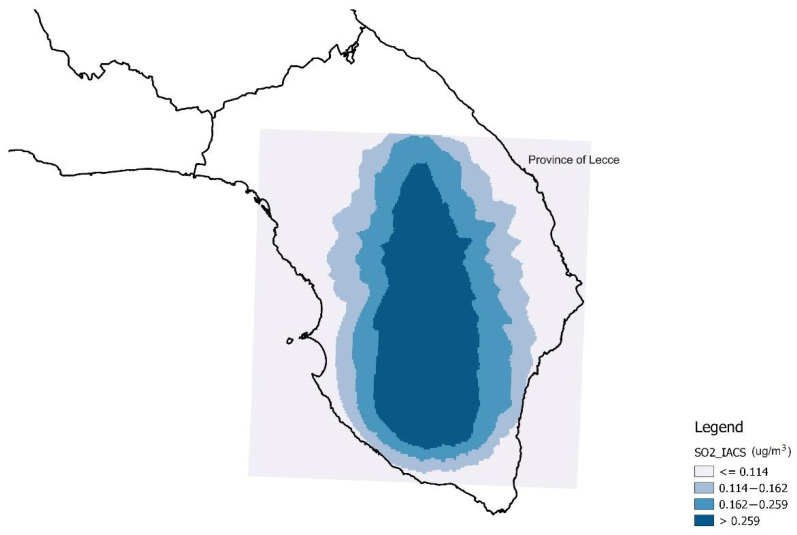
Exposure classes according to the quartiles of the sulfur dioxide distribution of the Industrial Area of Central Salento (IACS) (SO_2__IACS), attributed to georeferenced subjects (values in µg/m^3^).

**Figure 4:**
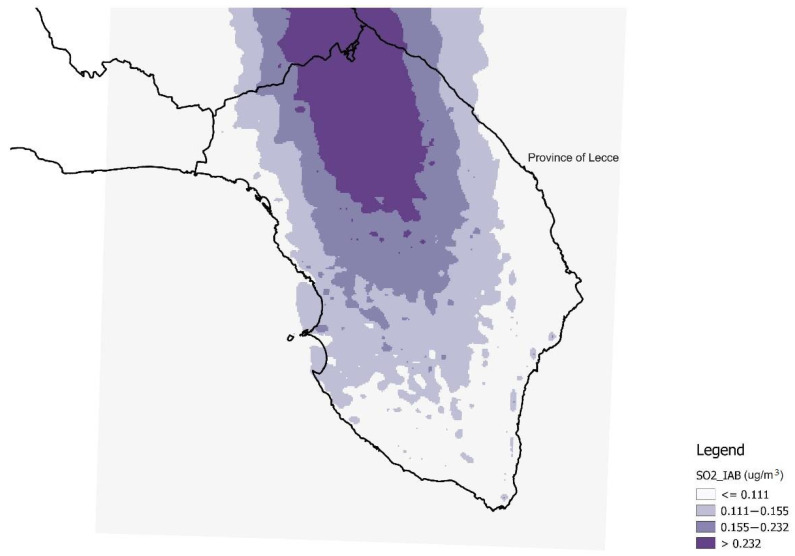
Exposure classes according to the quartiles of the sulfur dioxide distribution of the Industrial Area of Brindisi (IAB) (SO_2__IAB), attributed to georeferenced subjects (values in µg/m^3^).

In the multiple regression analyses, expSO_2__IACS and expSO_2__IAB were also considered categorically, according to four classes of exposure. Figure 3 and Figure 4 report the four classes of exposure to IACS and IAB, defined as quartiles of the distributions of expSO_2__IACS and expSO_2__IAB, respectively, attributed to the georeferenced subjects.

The study also considered exposure to other air pollution sources in the residential setting. These exposures were deduced from the answers to the questions in the questionnaire about the possible presence of pollution sources in the proximity of the residences, such as chemical/petrochemical plants, thermoelectric plants, port, activities with the potential presence of asbestos (shipyard, warehouses covered with asbestos, railway rolling stock production/repair plants), proximity to asbestos artefacts (such as asbestos roofing, asbestos water tanks, asbestos flues, and in general buildings containing asbestos), quarries or mines, incinerators, and landfills. Indoor exposure to fumes potentially originating from wood-fired fireplaces was also considered.

The occupational exposure was defined as the prevalent activity declared in the questionnaire (carried out for at least 10 years from 2007) in potentially critical sectors (chemistry, construction, wood and similar, metallurgy, mining, agriculture defined according to the specific codes set by National Institute for Occupational Accident Insurance (INAIL)). Occupational exposure to hazardous substances was defined considering the declared exposure to solvents, paints, abrasives, silica, cement, asbestos, fibrous material, physical agents/radiation, and chemicals in potentially critical sectors.

#### 2.3.3. Exploratory Analysis to Assess Radon Exposure 

The limited human and financial resources available, as well as the timing of the project, made it possible to measure radon concentrations in only 78 houses belonging to 37 cases and 41 controls, thus performing a preliminary explorative assessment of the local burden of this risk factor. For this reason, radon exposure was not considered in the main analysis, but it was presented as an additional exploratory analysis. The houses of cases and controls were randomly selected among those frequently inhabited in the last 20 years that have not undergone significant structural interventions. Dosimeters for the passive measurement of radon concentration (model CR39, produced by Miam Ltd., Piacenza, Italy) were placed in the bedrooms for 6 months in autumn/winter seasons. The dosimeters were subsequently replaced with new ones for an additional six months during the spring/summer seasons, in order to evaluate the seasonal variability of radon concentrations.

### 2.4. Statistical Analysis

Descriptive analyses were carried out by comparing the frequency distributions of the cases and controls according to the factors considered through contingency tables. The statistical significance of the differences was assessed using the chi-square test or Fisher’s exact test if the number of observations was <5. For continuous variables, the differences between the means of the cases and controls were evaluated using the Student’s *t*-test.

To assess the role of the factors considered jointly with respect to the presence of LC (cases) or absence of LC (controls), a multiple regression analysis was performed considering the dichotomous outcome defined as the presence/absence of LC (case–control) to be the dependent variable and those factors resulted significant in the descriptive analyses and other variables reported in the literature as risk factors for LC (socio/demographic status, lifestyle habits, clinical history, residential and occupational exposure) To be the independent variables.

Depending on the selected factors, the LC risk associations were estimated by Odds Ratio (OR) calculated through a conditional multiple logistic regression model, this being a case–control study. The variables entered in the linear predictor of the model are not collinear. The ORs of each factor were adjusted for the influence of the other factors analyzed. Each OR is accompanied by the Confidence Interval at 90% of probability (90% CI). We chose to use a wider confidence interval as we wanted to be more protective against the null hypothesis as typically used in epidemiological surveillance [42]. Consequently, the statistical significance threshold was set for *p* < 0.1. In agreement with recent studies to move beyond the concept of statistically significance [43], both statistically significant results and others for which only signals of association exist were reported in the Results section.

Moreover, an in-depth analysis was carried out for each of the categorical variables of environmental exposure such as expSO_2__IACS; expSO_2__IAB; and the dichotomous exposures, obtained from the questionnaire, to incinerators, landfills, quarries, asbestos artefacts, activities with the potential presence of asbestos, adjusting for individual factors (body size, education, marital status, consumption of red meat, alcohol consumption and smoking habits) and occupational exposure.

Finally, an ad hoc analysis on the carcinogenic effects of the use of pesticides without the use of protective devices, adjusting for the previously listed adjustment variables, was carried out.

### 2.5. Ethical Aspects

The study was approved by the Ethics Committee of the ASL of Lecce (Registration number 242/16), responsible for the study area. Participation in the study was voluntary, and no incentives were offered. All participants received written and oral information about the study and signed the informed consent form for research purposes. All data were collected and analyzed in accordance with the Italian Law n. 196 of 30 June 2003 (“protection of personal data”) and subsequent amendments, in full compliance with European directives about citizens’ privacy.

## 3. Results

### 3.1. Descriptive and Multiple Regression Analyses

On the basis of a 1:3 matching ratio, 442 cases and 1326 controls were enrolled. Table 3 shows the results of the descriptive analysis, and Table 4 shows the risk associations of LC.

Adenocarcinoma was the most frequent histotype of LC detected both in men (33.90%) and women (62.64%). Among both sexes, no difference in mean age was observed between the cases and controls (men: 71.0 ± 8.9 years vs. 70.4 ± 9.7 years; women: 65.9 ± 11.7 years vs. 65.9 ± 11.9 years) (Table 3). The cases among the men had significantly lower levels of education than among controls: 79.14% of men attended primary and lower secondary schools vs. 70.58% of controls (Table 3). Additionally, male individuals who graduated from middle school had a 41% decrease in LC risk compared to those with a lower education level (aOR = 0.59; 90%CI 0.40–0.86), while the risk decreased up to 53% in men who finished higher middle school (aOR = 0.47; 90%CI 0.31–0.73) (Table 4).

Conversely, 37.78% of female cases reached upper secondary or higher education vs. 24.16% of controls (Table 3), but no significant association was observed between level of education and risk of LC (Table 4). Men with LC were less likely to have a normal weight than the control group (47.58% of cases vs. 52.81% of controls) (Table 3), but in the multiple regression analysis, body size did not influence the LC risk (Table 4). About 25.07% of men reported engaging in irregular physical activity compared to 33.24% of controls (Table 3). Furthermore, the risk of LC increased by 88% in those who declared doing heavy work with no other physical activity compared to men who undertook heavy work and also at least 30 min of any physical activity 2 or 3 times per week (aOR = 1.88; 90%CI 1.31–2.72) (Table 4). In contrast, such association was not detected among women.

Men with LC were more likely to abuse alcohol than the control group (40.46% of cases vs. 33.72% of controls) (Table 3). The excessive use of alcohol among men and women increased the adjusted risk of LC by 22% and 144%, respectively, compared to normal use (men: aOR = 1.22; 90%CI 0.93–1.62; women: aOR = 2.44; 90%CI 1.01–5.94) (Table 4).

There were no statistically significant differences between cases and controls across both men and women with regard to excessive consumption of red meat (>2 times in a week) (Table 3). Furthermore, excessive meat intake was not significantly associated with an increased risk of LC in either sex (Table 4).

Only 2% of male cases were non-smokers (NS) (8 out of 351), and the proportion of both former smokers (FS) and current smokers (CS) was significantly higher among cases than controls (OR_FS + CS vs. NS_ = 18.8; 90%CI 10.33–34.26). Similar results were found for the ANPYC variable (62.7 ± 36.6 packages year vs.26.6 ± 30.4 packages year, p < 0.001) (Table 3). Based on multivariate analysis, the risk of LC increased by 3% (aOR = 1.03; 90%CI 1.03–1.04) for each increase in packages year of cigarettes (Table 4). In women, the percentages of CS and FS were higher among the cases (OR_CS + FS vs. NS_ = 4.98; 90%CI 3.09–8.02), as well as the average number of packages year of cigarettes smoked, but there were 34% of NS among the subjects with LC (31 out of 91), and the number of women exposed to second-hand smoke was also higher among cases than controls (Table 3). Moreover, the ANPYC was higher in women with cancer (22.7 ± 25.1 packages year vs. 5.9 ± 13.7 packages year, p < 0.001) (Table 3). As shown in Table 4, the risk of LC among women increased by 6% (aOR = 1.06; 90%CI 1.04–1.08) for each unitary of ANPYC. 

For both sexes, family history of any cancer was more frequent among cases than in controls (men: 47.86% vs. 35.42%; women: 51.65% vs. 45.05%) (Table 3). An association between LC and a family history of LC was observed both in male cases (28% risk increase compared to controls) and in female cases (81% risk increase compared to controls) (Table 4).

Among men with LC, a significantly higher numbers of cases than controls reported living near buildings that might contain asbestos (30.72% vs. 22.44%) (Table 3), but the multivariate analysis did not suggest any risk associations of LC with this exposure (Table 4). For women affected by LC, the results were comparable to those observed for men, albeit based on a small number of cases (30.77% vs. 24.90%) (Table 3), while the positive association between the residential proximity to buildings with the presence of asbestos and the onset of LC, although significant had a low degree of precision (aOR = 11.81; 90%CI 2.76–50.49) (Table 4).

According to the questionnaire responses, male cases with LC lived more frequently near an incinerator than controls (2.56% vs. 0.47%) (Table 3), and an increased risk of LC associated with this type of exposure was confirmed by multivariate analysis despite the limited number of cases (aOR = 9.83; 90%CI 2.45–39.44) (Table 4). Similarly, a greater number of cases compared to controls reported living near landfills (men: 7.41% vs. 4.27%; women 9.89% vs. 5.13%) (Table 3), but no significant association was found between LC and this exposure (Table 4).

The exposure to IAB (considering SO_2_ as a proxy) did not show significant differences between cases and controls (measured as mean concentration within each group) (Table 3), and this result was supported by the lack of risk association between exposure to IAB and LC (Table 4). Among men, cases were exposed to a significantly higher average concentration of SO_2_ produced by IACS than controls (0.363 ± 0.739 µg/m^3^ vs. 0.253 ± 0.428 µg/m^3^) (Table 3). A 142% increase in risk was observed in the group that was more exposed to IACS (4th quartile) compared to the reference 1st quartile (aOR = 2.42; 90%CI 1.58–3.72) (Table 4). In addition, a 21% increase in risk was associated with a 1 µg/m^3^ increase of SO_2_ levels, even though this association did not reach statistical significance (risk trend = 1.21; 90%CI 0.97–1.51). In contrast, no association was found between the risk of LC and exposure to SO_2_ produced by IACS among women.

In both men and women, there were no significant risk associations between occupational exposure and the onset of LC after adjusting for the other risk factors (Table 4).

On the other hand, the use of pesticides in agriculture without wearing personal protective equipment was more common among male cases (64.52% vs. 35.14) (Table 3), resulting in a statistically significant association with the occurrence of LC in men (426% increase in risk: aOR = 5.26; 90%CI 1.58–17.53) but not in women (Table 4).

The exploratory analysis highlighted a higher annual average concentration of radon, which was higher in the houses of cases than in those of controls, but this difference did not reach statistical significance (207 Becquerel/m^3^ vs. 192 Becquerel/m^3^; *p* = 0.667).

### 3.2. Sensitivity Analyses

A sensitivity analysis was conducted using an alternative methodological approach, i.e., to consider the exposures to the thermoelectric power plants and cement plants (it should be noted that expSO_2__IACS and expSO_2__IAB are related with each other even if not collinear), self-declared domestic exposures, and residential exposures separately. Analyses provided, for all exposures, risk estimates for LC through OR adjusted for education, marital status, physical activity, alcohol consumption, active and passive smoking, cancer familiarity, and occupational exposure. Sensitivity analyses confirmed an increased risk of LC for men who lived near an incinerator and for those who were more exposed to IACS. The association of increased risk for LC with proximity to asbestos artefacts with the potential presence of asbestos was also confirmed among women.

## 4. Discussion

This study explored the association between individual, occupational, and environmental risk factors and the incidence of LC in the province of Lecce (Salento, southern Apulia, Italy) through a case–control study. Few previous studies have investigated the relationship between health and the environment in the Salento area. A survey on the quality of groundwater in the “cluster area” highlighted the presence of heavy metals attributable to emissions from industrial sources [44]. A study on the occurrence of early biomarkers of LC in children living in this area [45] showed a prevalence of genotoxic effects in their buccal cells, which appeared lower if compared to children of other Italian areas suffering from heavy environmental impacts [46] but higher than those living in areas with low anthropogenic pressures [47], thus indicating a possible exposure to environmental or behavioral factors (i.e., second-hand smoke, diet, overweight).

From the analysis of self-reported exposures to environmental factors collected through the questionnaire, we observed a significant association between alcohol consumption and a 2.4-fold increased risk of LC in female cases, although this relationship remains controversial [22]. In line with our findings, Korte and co-authors (2002) reported a 234% adjusted risk increase for smoking in the highest alcohol consumption category (≥2000 g ethanol per month) in women compared to non-drinkers [48]. Recently, a systematic review conducted on the findings of the European Prospective Investigation into Cancer (EPIC) and Nutrition study, based on one of the largest cohorts worldwide, did not find any association between mean lifetime ethanol intake and LC [49].

Regarding the excessive consumption of red meat, no significant association was observed with the risk of LC, which is consistent with the most recent data. In a population-based prospective cohort study conducted in Japan on 73,187 participants aged 45–74 years, total red meat intake was linked to an increase of risk of 25% for LC in men, but no positive association was observed among women [50]. Furthermore, according to a systematic review of 100 high-quality studies based on the EPIC cohort, no significant association between meat consumption and risk of LC can be observed [49].

In this study, the vast majority of LC cases were in subjects reporting smoking cigarettes, with adenocarcinoma the most frequent histological subtype, especially among women. This result is in line with data which showed that approximately 40 to 60% of LC in women correspond to adenocarcinoma since 1960 [51], thus suggesting that other concurrent factors are involved in exponentially increasing LC risk in smokers. Additionally, the observed increased rate of LC among non-smoker women is indicative of the possible existence of other etiological factors in addition to smoking which include second-hand smoke or/and environmental exposures [52]. Among women, we found a greater risk of LC among those exposed to secondhand smoke and those with family history of LC. Previous studies evaluating the relationship between passive smoking and LC risk in women have produced contradictory results, mainly because of inaccuracies in the self-reporting of passive exposures and the consequent misclassification of exposures [24]. According to a retrospective study carried out in Morocco, among 101 women with diagnosis of LC, the percentage of non-smokers was 75%, whereas 14% of patients were exposed to environmental tobacco smoke [53]. In contrast, a prospective cohort study on 76,304 postmenopausal women found that, among non-smokers, ever-exposed passive smokers did not present an increased risk of LC [24].

In this study, family history was also associated with an increase of LC in both men and in women, as recognized by substantial evidence [54]. A meta-analysis based on 41 studies showed that a family history of LC was associated with a 1.5-fold and a 1.73-fold higher risk of LC among men and women, respectively [55]. A pooled analysis including 24 case–control studies found a significant relationship between family history of LC and occurrence of LC, but, after adjustment for age, sex, ethnicity, education, smoker type and pack years, a similar pattern was seen in both sexes [11].

An excess risk was observed in the study area among women, but not in men, living in the proximity of activities with the potential presence of asbestos. In a pooled analysis of case–control studies including 17,705 LC cases and 21,813 controls, exposure to asbestos, even adjusted for tobacco smoking, resulted in an increased risk for LC by 24% in men and 12% in women [56]. However, this estimated LC risk was related to occupational asbestos exposure, whereas our study evaluated the occurrence of LC in case and controls obtained from the general population, and, in both studies, misclassifications of exposures may have occurred.

An excess of risk of LC was observed among men who declared that they lived near an incinerator, although the estimates were based on a low number of subjects. Overall, a scarce number of studies investigated the relationship between exposure to incinerator emissions and risk of LC. An Italian study applying a modified risk-assessment model to estimate the LC risk related to pollutants emitted from an incinerator plant found that the maximum risk of developing LC occurred closer to the stack of the plant although this risk excess was below the WHO target risk range [57]. As for the results relating to asbestos exposures, also for those relating to residential exposure to emissions from incinerators, it is important to clarify that they may be affected by inaccuracies, due to the small number of cases and the possibility of recall bias. For this reason, the results on the association of LC with environmental exposures through proxies of exposures, such as activities close to the potential presence of asbestos and incinerators, generate hypotheses to be further investigated with validated exposure measurements.

IARC has classified outdoor air pollution as carcinogenic to humans and concluded that there is sufficient evidence that exposure to outdoor air pollution causes LC [58,59,60], exploiting a huge body of epidemiological evidence [61,62]. The atmospheric emissions associated with cement production include several compounds, the most common being NO_x_, SO_2_, particulate matter, carbon monoxide, and carbon dioxide, as well as small quantities of the volatile organic compounds, ammonia, chlorine, and hydrogen chloride [63,64]. Our results show an excess of risk of LC among men exposed to the highest levels of SO_2_ emitted in Industrial Area of Central Salento (used as a surrogate of emissions in the study area) if compared to those less exposed. Although in the present study this association occurs only among men, a synergistic effect between environmental and occupational exposures and smoking habits cannot be excluded. A systematic review and meta-analysis including 26 occupational cohort- and case–control studies did not find adequate evidence for increased risk of any cancers associated to cement exposure [63]. On the other hand, another systematic review of 26 studies on cement production workers and non-exposed or low-exposed subjects, showed reduced lung function levels (e.g., respiratory symptoms, asthma, chronic bronchitis, chronic obstructive pulmonary disease) to exclusively be a consequence of high exposures to total and respiratory dust. However, the lack of adjustment for possible confounders and other methodological issues were the main limitations of most included studies [65]. Finally, a recent systematic review evaluating the effects of cement-plant emissions on the general population health on a total of 24 studies, reported positive associations between living nearby a cement plant and respiratory symptoms or diseases and—in some studies—an increased risk of LC in the exposed compared to the unexposed subjects [66].

Multiple occupational agents/activities, including mining and usage of asbestos, exposure to metals and other chemicals, exposure to silica dust and radiation, have been linked to LC [67]. However, studies of LC in occupational populations are often characterized by a small sample size and an inability to control for interactions with tobacco smoking [68]. In this study, we detected no relationship between occupational exposures and LC risk but, on the other hand, we noted a marked association of LC with exposures in agriculture settings among men who did not wear personal protective equipment. This result requires an in-depth analysis through a larger sample and improving the exposure assessment, e.g., using job-exposure matrices. Previous studies evaluating exposure to specific pesticides among male pesticide applicators with the highest exposure category of lifetime reported inconsistent results in relation to occurrence of LC, possibly due to exposure misclassification and residual confounders, including second-hand smoking, not evaluated in the statistical models [69,70,71].

The observation of rather high average radon concentration values, particularly among cases, reinforces the need to strengthen the measurements.

Regarding radon (which has been recently acknowledged as an underestimated problem in the study area (Maggiore et al., 2020)), despite having applied a robust methodology based on single measurements in the houses of cases and controls, the small number of measurements (and therefore the low power of the exploratory analysis) did not allow us to confirm the risk association of LC with radon reported by Ferri et al. (2018). For these reasons, it will be useful to increase the number of measurements of radon and possibly to perform a Health Impact Assessment with the aim of analyzing all emissions (including those from plants not subject to the IEA) from both the industrial areas of Galatina–Soleto and the district of Maglie in the “cluster area” [72].

This study presented several strengths. Firstly, it is a case–control study, which represents one of the most advanced analytical designs used in environmental epidemiology, as well as being particularly suitable for the study of multiple risk factors relating to rare diseases with a long induction period such as LC. In particular, this type of study allows researchers to evaluate the exposure both to specific environmental pollutants and to individual risk factors through the administration of questionnaires. Secondly, cases and controls were matched by age, deleting the confounding effect of age, while controls were patients enrolled from the same hospital as the cases in order to reduce errors both in control selection and in collected information. In addition, the study estimated individual exposures to industrial emissions through pollutant diffusion models, significantly reducing the misclassification of exposures compared to the use of distance as a proxy measure.

Nonetheless, due to the lower incidence of LC among women, about a quarter fewer women than men were recruited, resulting in less statistical power, which in turn may have reduced the accuracy of the findings. Moreover, considering the long latency period between exposure and the effect, the “body size” and lifestyle variables were not traced back to the relevant exposure periods. In particular, recall bias may represent a limitation in retrospective studies that use a case–control design, as ours did, since it could lead to the misclassification of various types of past exposures to a similar or different extent in cases and controls (non-differential and differential misclassification, respectively), resulting in biased risk estimates.

In this study environmental exposures were based solely on residence, regardless of the time in a day spent away from home for personal and work reasons. The presence of other industrial plants in the industrial area, in addition to those subject to environmental impact assessment, may lead to a non-differential misclassification of exposure, resulting in a wrong estimation of the risk, more likely in the direction of underestimation.

The assumption that the diffusion pattern estimated through a single reference year for both emissions (1990 for IACS, 1993 for IAB) and meteorology (2005 for IACS and 2007 for IAB) is representative of the distribution of emissions over the entire period of residence considered may be weak. However, in order to mitigate this limitation, we have to consider that the meteorological characteristics and emission data did not show significant changes during the residential exposure period, and therefore the exposure pattern during the same period may not have significantly changed.

Residential exposure to SO_2_ was considered as a proxy of exposure of IACS (SO_2_ strongly correlated with NO_x_), thus assuming that other pollutants such as NO_x_ could be associated with the resulting risks. These estimates were annual averages, not including short-term concentration peaks, which could cause higher exposures. In fact, we considered it appropriate to analyze the risk of cancer in potentially more-exposed subjects (i.e., those residing in areas closer to the sources of pollution) than those who were potentially less exposed (i.e., individuals residing in areas further away from the sources of pollution), adding to this result the LC risk with increasing SO_2_ concentration (it should not be considered as a dose–response relationship but as a more reliable proxy of exposure to IACS). The analysis based on proxies of exposure does not allow us to exclude the residual confounding due to other factors that contribute to the characterization of the exposure of the same area.

Finally, this study enables us to assess the overall effect of a wide range of risk factors (environmental exposures, occupational settings, individual socio-demographic information, lifestyle, smoking habits, and diet) through multiple conditional logistic regression models. Considering the number of variables, this model was extremely complex, therefore other models that analyzed one factor at a time were applied. The final considerations were drawn by evaluating the consistency of the results obtained with the different study methodologies starting from the descriptive analyses up to the comparison with more complex models.

## 5. Conclusions

The excess risk of LC evidenced among both men and women suggests that, in addition to the expected strong role of cigarette smoking, concomitant exposures to additional environmental, occupational, and lifestyle factors should be explored more thoroughly, especially occupational exposure and exposure to asbestos, radon, and other environmental pollutants emitted by the Industrial Area of Central Salento located in the “cluster area” for LC. To this end, the authors suggest continuing the study with a case–control surveillance program in which the recruitment of cases and controls is prolonged in order to: (i) increase the number of observations (together with statistical power, especially in women); (ii) increase radon measurements, so that they can become representative of the exposure of the area under study; (iii) enhance the accuracy and precision of environmental and occupational exposures; and (iv) design a specific epidemiological survey focused on the “cluster area” due to the specific exposures and risks emerged by this comprehensive study. These objectives should be achieved through an in-depth integrated assessment of the environmental impact on health.

## Figures and Tables

**Table 1:** Variables selected for the statistical analyses.

Variables Selected for Each Section	Description	Type
**1—PERSONAL INFORMATION**		
**Age**	Age at the interview	Continuous
**Schooling**	4 categories	Ordinal
	Elementary school or no degree	
	Middle school license	
	Higher middle school license	
	Graduation	
**Marital status**	2 categories	Dichotomous
	Married/Cohabiting	
	Single, separated, divorced, widower	
**Body size**	Self-declaration of body size (4 categories)	Ordinal
	Underweight	
	Normal weight	
	Overweight	
	Obesity	
**2—BEHAVIOURS AND LIFESTYLES**		
**Physical activity**	Self-declaration of the type of physical activity that may be practiced (3 categories)	Nominal
	Active (hard work + other physical activity)	
	Hard work without other physical activity	
	Sedentary work and no physical activity	
**Excessive alcohol consumption**	Self-declaration of consumption of more than 2 glasses of alcohol per day	Dichotomous
	Yes	
	No	
**Excessive meat consumption** (>2 times in a week)	Self-declaration of consumption of more than 2 times in a week	Dichotomous
Yes	Yes	
No	No	
**Smoking habits**	Self-declaration of smoking habits in 3 categories	Nominal
Smoker	Smoker	
Former smoker	Former smoker	
No smoker	Non-smoker	
**ANPYC** Average Number of Packages Year of Cigarette	self-declaration of number of cigarettes smoked per day ANPYC = (average number of cigarette per day × years of active smoking)/20	Continuous
**Exposure to second hand smoke**	Self-declaration of exposure to second-hand smoke	Dichotomous
Yes	Yes	
No	No	
**3—PERSONAL AND FAMILY CLINICAL HISTORY**		
**Positive family history for cancer**	at least one family member including mother/father, brother/sister, grandfather/grandmother	Dichotomous
	Yes	
	No	
**4—EXPOSURE TO SOURCES OF AIR POLLUTION**		
**Frequent use of the wood-fired fireplace?**	Self-declaration refers to the house in which the subject lived for at least 10 continuous years before 2006	Dichotomous
	Yes	
	No	
**Presence of asbestos artefacts nearby?**	Self-declaration refers to the house in which the subjects lived for at least 10 continuous years before 2006. Artefacts such as asbestos roofing, asbestos water tanks, asbestos flues, and in general buildings containing asbestos	Dichotomous
	Yes	
	No	
**In the vicinity of the residence, presence of**	Self-declaration refers to the house in which the subjects lived for at least 10 continuous years before 2006.	
Intense traffic		Dichotomous
Chemical/petrochemical plant		Dichotomous
Thermoelectric power plant		Dichotomous
Port		Dichotomous
Industrial Area		Dichotomous
Activities with potential presence of asbestos	As a shipyard, sheds covered with asbestos, railway rolling stock production/repair plants	Dichotomous
Quarrying or mining		Dichotomous
Incinerator		Dichotomous
Landfill		Dichotomous
**SO_2_ exposure from industrial plants**	Data from dispersion models of SO_2_	
expSO_2__IACS	Individual exposure to the Industrial Area of Central Salento (IACS), defined on the basis of the SO_2_ distribution produced by IACS (emission data from 1990; meteorological data from 2005)	Continuous
expSO_2__IAB	Individual exposure to the Industrial Area of Brindisi (IAB), defined on the basis of the SO_2_ distribution produced by the IAB (emission data from 1993; meteorological data from 2005)	Continuous
**6—WORK AND PROFESSIONAL HISTORY**		
**Occupational exposure to hazardous substances**	Hazardous substances such as solvents, paints, abrasives, silica, cement, asbestos, fibrous material, physical agents/radiation and chemical substances in potentially critical production sectors (chemistry, construction, wood and similar, metallurgy, mining, agriculture) defined according to the National Institute for Occupational Accident Insurance code.	Dichotomous
	Yes	
	No	
**Prevalent activity (≥10 years, accrued by 2007) in potentially critical sector**	Prevalent activity declared in the questionnaire (carried out for at least 10 years from 2007) in potentially critical sectors (chemistry, construction, wood and similar, metallurgy, mining, agriculture) defined according to the National Institute for Occupational Accident Insurance code	Dichotomous
	Yes	
	No	
**Prevalent activity (≥10 years, accrued by 2007) in the agricultural sector using plant protection products with no personal protection equipment**	Prevalent activity declared in the questionnaire (carried out for at least 10 years from 2007) in the agricultural sector using plant protection products with no personal protection equipment	Dichotomous
	Yes	
	No	

**Table 2:** Subjects’ exposure distribution for exposure to sulfur dioxide (SO_2_) emitted by both Industrial Area of Central Salento (IACS) and Industrial Area of Brindisi (IAB).

SO_2_ (µg/m^3^)	*n*	Mean	SD	p25	p50	p75	p90	min	max
expSO_2__IACS	1703	0.276	0.528	0.114	0.162	0.259	0.467	0.020	5.378
expSO_2__IAB	1703	0.183	0.093	0.111	0.155	0.232	0.316	0.042	0.551

*Legend: exp_SO_2__IACS: individual exposure to the atmospheric pollution produced by plants included in the IACS; exp_SO_2__IAB: individual exposure to the atmospheric pollution produced by plants included in the IAB; n: number of subjects; SD: standard deviation; p25: 25th percentile; p50: 50th percentile; p75: 75th percentile; p90: 90th percentile; min: minimum value; max: maximum value.*

**Table 3:** General characteristics of the population under study.

	Men (*n* = 1404)			Women (*n* = 364)		
Variables	Cases [*n* (%)]	Controls [*n* (%)]	*p*-Value	Cases [*n* (%)]	Controls [*n* (%)]	*p*-Value
**Classifications of pulmonary neoplasms**						
Adenocarcinoma	119 (33.90)		<0.001 ^#^	57 (62.64)		<0.001 ^#^
Squamous carcinoma	99 (28.21)			7 (7.69)		
Small cell carcinoma	40 (11.40)			6 (6.59)		
Less frequent neoplasms ç	51 (14.53)			13 (14.29)		
Other çç	42 (11.97)			8 (8.79)		
**Age** (mean ± Standard Deviation)	351	1053		91	273	
	71.00 ± 8.90	70.40 ± 9.70	0.241	65.9 ± 11.70	65.9 ± 11.90	0.962
**Schooling**	350	1040		90	269	
Elementary school or no degree	186 (53.14)	409 (39.33)	<0.001	43 (47.78)	133 (49.44)	0.032
Middle school license	91 (26.00)	325 (31.25)		13 (14.44)	71 (26.39)	
Higher middle school license	56 (16.00)	247 (23.75)		23 (25.56)	42 (15.61)	
Graduation	17 (4.86)	59 (5.67)		11 (12.22)	23 (8.55)	
**Marital status**	349	1051		90	270	
Married/Cohabiting	287 (82.23)	921 (87.63)	0.011	62 (68.89)	187 (69.26)	0.947
Single, separated, divorced, widower	62 (17.77)	130 (12.37)		28 (31.11)	83 (30.74)	
**Physical activity**	351	1053		91	273	
Active (hard work + other physical activity)	88 (25.07)	350 (33.24)	<0.001	28 (30.77)	66 (24.18)	0.386
Hard work without other physical activity	169 (48.15)	346 (32.86)		23 (25.27)	67 (24.54)	
Sedentary work and no physical activity	94 (26.78)	357 (33.90)		40 (43.96)	140 (51.28)	
**Body size**	351	1051		91	273	
Underweight	118 (33.62)	306 (29.12)		36 (39.56)	95 (34.80)	
Normal weight	167 (47.58)	555 (52.81)	0.006	46 (50.55)	136 (49.82)	0.264
Overweight	53 (15.10)	177 (16.84)		9 (9.89)	32 (11.72)	
Obesity	13 (3.70)	13 (1.24)		<3	10 (3.66)	
**Excessive alcohol consumption** (>2 glasses of alcohol per day)	351	1044		91	273	
Yes	142 (40.46)	352 (33.72)	0.022	11 (12.09)	34 (12.45)	0.927
No	209 (59.54)	692 (66.28)		80 (87.91)	239 (87.55)	
**Excessive meat consumption** (>2 times in a week)	351	1053		91	273	
Yes	279 (79.49)	845 (80.25)	0.758	72 (79.12)	193 (70.70)	0.118
No	72 (20.51)	208 (19.75)		19 (20.88)	80 (29.30)	
**Smoking habits**	351	1053		91	273	
Smoker	82 (23.36)	207 (19.66)	<0.001	15 (16.48)	37 (13.55)	<0.001
Former smoker	261 (74.36)	522 (49.57)		45 (49.45)	48 (17.58)	
No smoker	8 (2.28)	324 (30.77)		31 (34.07)	188 (68.86)	
**ANPYC ^$^ (*n*)** mean ± Standard Deviation (Packs/year)	351	1053		91	273	
	62.7 ± 36.60	26.6 ± 30.40	<0.001	22.7 ± 25.10	5.9 ± 13.70	<0.001
**Exposure to second hand smoke**	351	1053		91	273	
Yes	125 (35.61)	315 (29.91)	0.046	65 (71.43)	159 (58.24)	0.025
No	226 (64.39)	738 (70.09)		26 (28.57)	114 (41.76)	
**Positive family history for cancer** ^§^	351	1053		91	273	
Yes	168 (47.86)	373 (35.42)	<0.001	47 (51.65)	123 (45.05)	0.275
No	183 (52.14)	680 (64.58)		44 (48.35)	150 (54.95)	
**Frequent use of the wood-fired fireplace? °**	351	1053		91	273	
Si	225 (64.10)	726 (68.95)	0.093	60 (65.93)	183 (67.03)	0.847
No	126 (35.90)	327 (31.05)		31 (34.07)	90 (32.97)	
**Presence of asbestos artefacts nearby? °^**	306	949		78	241	
Yes	94 (30.72)	213 (22.44)	0.003	24 (30.77)	60 (24.90)	0.306
No	212 (69.28)	736 (77.56)		54 (69.23)	181 (75.10)	
**In the vicinity of the residence, presence of °**	351	1053		91	272	
Intense traffic	53 (15.10)	182 (17.28)	0.591	13 (14.29)	48 (17.65)	0.495
Chemical/petrochemical plant	4 (1.14)	4 (0.38)	0.101	<3	<3	nc
Thermoelectric power plant	<3	6 (0.57)	nc	<3	<3	nc
Port	4 (1.14)	7 (0.66)	0.382	<3	<3	nc
Industrial Area	19 (5.41)	67 (6.36)	0.521	10 (10.99)	28 (10.26)	0.843
Activities with potential presence of asbestos *	22 (6.27)	50 (4.75)	0.264	10 (10.99)	9 (3.30)	0.004
Quarrying or mining	18 (5.13)	34 (3.23)	0.103	4 (4.40)	3 (1.10)	0.047
Incinerator	9 (2.56)	5 (0.47)	0.001	<3	<3	nc
Landfill	26 (7.41)	45 (4.27)	0.020	9 (9.89)	14 (5.13)	0.106
**SO_2_ exposure from industrial plants**						
expSO_2__IACS ** (*n*)	333	1021		86	263	
mean ± Standard Deviation (µg/m^3^)	0.363 ± 0.739	0.253 ± 0.428	<0.001	0.259 ± 0.389	0.259 ± 0.588	0.995
expSO_2__IAB *** (*n*)	333	1021		86	263	
mean ± Standard Deviation (µg/m^3^)	0.187 ± 0.097	0.184 ± 0.094	0.596	0.179 ± 0.092	0.177 ± 0.088	0.855
**Prevalent activity (≥10 years, accrued by 2007) in potentially critical sector ^@^**	351	1053		91	273	
Yes	182 (51.85)	485 (46.06)		12 (13.19)	70 (25.64)	
No	169 (48.15)	568 (53.94)	0.06	79 (86.81)	23 (74.36)	0.014
**Occupational exposure to hazardous substances** ****	351	1053		91	273	
Yes	95 (27.07)	301 (28.58)	0.584	<3	21 (7.69)	nc
No	256 (72.93)	752 (71.42)		89 (97.80)	252 (92.31)	
**Prevalent activity (≥10 years, accrued by 2007) in the agricultural sector using plant protection products with no personal protection equipment**	31	74		<3	<3	
Yes	20 (64.52)	26 (35.14)	0.006	<3	<3	nc
No	11 (35.48)	48 (64.86)		<3	<3	

Legend—n: number of subjects; nc: not calculable; ^ç^: *neuroendocrine, atypical and carcinoid tumors;* ^çç^: *heteroplasias not histotyped or with histological/cytological examination not available;* ^$^: *Average Number of Packages Year of Cigarette smoked (ANPYC);* ^§^: *at least one family member including mother/father, brother/sister, grandfather/grandmother;* ^: *artefacts such as asbestos roofing, asbestos water tanks, asbestos flues, and in general buildings containing asbestos;* °: *the data refer to the house in which the subjects lived for at least 10 continuous years before 2006;* IACS: Area of Central Salento; IAB: Industrial Area of Brindisi; *: *shipyard, sheds covered with asbestos, railway rolling stock production/repair plants*; **: individual exposure to the IACS (1990 data), defined on the basis of the SO_2_ distribution produced by IACS; ***: individual exposure to the IAB (1993 data), defined on the basis of the SO_2_ distribution produced by the IAB that impacts part of the territory under study of the province of Lecce; ^@^: potentially critical production sectors: chemistry, construction, wood and similar, metallurgy, mining, and agriculture defined according to the National Institute for Occupational Accident Insurance (INAIL—Istituto Nazionale Assicurazioni Infortuni sul Lavoro) code; ****: solvents, paints, abrasives, silica, cement, asbestos, fibrous material, physical agents/radiation, and chemical substances in potentially critical production sectors. Note—^#^: *p*-value is referred to the comparison between the proportions of tumor types among cases, both in men and women.

**Table 4:** Conditional logistic multiple regression analysis of lung cancer risk as a function of socio-demographic factors, lifestyles, disease history, and residential and occupational exposures.

Variables	Variables Coding	aOR	*p*	90%CI	aOR	*p*	90%CI
		Men			Women		
**Schooling**							
*Reference class: e* *lementary school or no degree*	Middle school license	0.59	0.021	0.40–0.86	0.52	0.209	0.22–1.22
	Higher middle school license	0.47	0.005	0.31–0.73	2.08	0.140	0.92–4.72
	Graduation	0.77	0.540	0.39–1.54	1.12	0.878	0.34–3.67
**Marital status**							
*Reference class: Married or cohabiting*	Single, separated, divorced, widower	0.68	0.098	0.46–1.00	1.99	0.093	1.01–3.92
**Physical activity**							
*Reference class: active (hard work + other physical activity)*	Hard work without other physical activity	1.88	0.005	1.31–2.72	0.77	0.607	0.33–1.78
	Sedentary work and no physical activity	0.97	0.905	0.67–1.41	0.56	0.185	0.27–1.15
**Body size**							
*Reference class: normal weight*	Under weight	1.08	0.699	0.77–1.51	0.53	0.155	0.25–1.10
	Overweight	0.72	0.192	0.48–1.09	0.97	0.965	0.36–2.67
	Obesity	2.49	0.105	0.98–6.28		nc	
**Alcohol consumption**							
Excessive alcohol consumption (>2 glasses of alcohol per day)		1.22	0.232	0.93–1.62	2.44	0.099	1.01–5.94
**Red meat consumption**							
Excessive meat consumption (>2 times a week)		0.81	0.318	0.56–1.15	1.29	0.530	0.66–2.55
**Smoking habits**							
ANPYC ^$^	Unit increase of packets year of cigarettes	1.03	<0.001	1.03–1.04	1.06	<0.001	1.04–1.08
Passive smoke		1.04	0.820	0.77–1.41	1.75	0.148	0.93–3.30
**Positive family history for cancer** ***^§^***		1.28	0.149	0.97–1.69	1.81	0.094	1.01–3.24
Sources of atmospheric pollution inside or in the proximity of the residence *							
Presence of wood-fired fireplace		0.82	0.291	0.60–1.12	0.87	0.716	0.45–1.67
Asbestos artefacts ^		1.30	0.205	0.92–1.82	1.20	0.685	0.57–2.55
Intense traffic		0.93	0.763	0.63–1.37	0.73	0.546	0.31–1.73
Chemical/petrochemical plant		5.31	0.179	0.69–40.91	nc		
Activity with potential presence of asbestos ^#^		1.15	0.742	0.58–2.27	11.81	0.005	2.76–50.49
Quarrying or mining		0.82	0.666	0.38–1.76	3.90	0.231	0.60–25.26
Incinerator		9.83	0.007	2.45–39.44	1.57	0.765	0.13–19.04
Landfill		1.42	0.348	0.77–2.63	0.70	0.599	0.23–2.15
**SO_2_ exposure from industrial plants (values in µg/m^3^)**							
IACS **	2nd quartile (0.115–0.162)	1.18	0.498	0.79–1.78	0.74	0.532	0.34–1.62
*Reference class: 1st quartile*	3rd quartile (0.162–0.259)	1.08	0.760	0.71–1.64	1.30	0.582	0.60–2.82
	4th quartile (>0.259)	2.42	0.001	1.58–3.72	1.25	0.667	0.54–2.90
	trend for increase of 1 µg/m^3^	1.21	0.162	0.97–1.51	0.91	0.734	0.58–1.43
IAB ***	2nd quartile (0.115–0.115)	0.57	0.031	0.37–0.88	0.63	0.397	0.25–1.55
*Reference class: 1st quartile*	3rd quartile (0.115–0.232)	0.55	0.017	0.37–0.83	1.75	0.290	0.73–4.16
	4th quartile (>0.232)	1.49	0.110	0.99–2.26	0.62	0.348	0.27–1.42
	trend for increase of 1 µg/m^3^	2.67	0.298	0.57–12.60	0.23	0.468	0.01–6.36
**Occupational exposure**							
Prevalent activity (≥10 years, accrued by 2007) in potentially critical sector ^@^		1.08	0.727	0.75–1.56	0.39	0.067	0.17–0.91
Occupational exposure to hazardous substances ****		0.76	0.158	0.55–1.05	0.27	0.175	0.06–1.32
Prevalent activity (≥10 years, accrued by 2007) in the agricultural sector using plant protection products with no personal protection equipment.		5.26	0.023	1.58–17.53	nc		

Legend—aOR: adjusted Odds Ratio; p: *p*-value; 90%CI: Confidence Interval at 90% of probability; nc: not calculable; *^$^: Average Number of Packages Year of Cigarette smoked (ANPYC); ^§^: at least one family member including mother/father, brother/sister, grandfather/grandmother; ^: artefacts such as asbestos roofing, asbestos water tanks, asbestos flues, and in general buildings containing asbestos;* ^#^: shipyard, sheds covered with asbestos, railway rolling stock production/repair plants; ^§^: solvents, paints, abrasives, silica, cement, asbestos, fibrous material, physical agents/radiation and chemical substances; IACS: Area of Central Salento; IAB: Industrial Area of Brindisi; *: these data refer to the residence where subjects continuously lived *for at least 10 years before 2006;* **: individual exposure to the IACS (1990 data), defined on the basis of the SO_2_ distribution produced by IACS; ***: individual exposure to the IAB (1993 data), defined on the basis of the SO_2_ distribution produced by the IAB and which impacts part of the territory under study of the province of Lecce; ^@^: potentially critical production sectors: chemistry, construction, wood and similar, metallurgy, mining, and agriculture defined according to the National Institute for Occupational Accident Insurance (INAIL—Istituto Nazionale Assicurazioni Infortuni sul Lavoro) code; ****: solvents, paints, abrasives, silica, cement, asbestos, fibrous material, physical agents/radiation, and chemical substances in potentially critical production sectors.
